# Gd-Complexes of New Arylpiperazinyl Conjugates of DTPA-Bis(amides): Synthesis, Characterization and Magnetic Relaxation Properties

**DOI:** 10.3390/molecules20057807

**Published:** 2015-04-29

**Authors:** Abdullah O. Ba-Salem, Nisar Ullah, M. Nasiruzzaman Shaikh, Mohamed Faiz, Zaheer Ul-Haq

**Affiliations:** 1 Chemistry Department, King Fahd University of Petroleum and Minerals, Dhahran-31261, Saudi Arabia; E-Mail: abdullahbasalem@gmail.com; 2 Center of Research Excellence in Nanotechnology, King Fahd University of Petroleum and Minerals, Dhahran-31261, Saudi Arabia; 3 Physics Department, King Fahd University of Petroleum and Minerals, Dhahran-31261, Saudi Arabia; E-Mail: mmfaiz@kfupm.edu.sa; 4 Dr. Panjwani Center for Molecular Medicine and Drug Research, International Center for Chemical and Biological Sciences, University of Karachi, Karachi 75270, Pakistan; E-Mail: zaheer_qasmi@hotmail.com

**Keywords:** DTPA-bis(amide), arylpiperazine, Gd(III)-complexes, MRI contrast agents, relaxivity, magneto-pharmaceuticals

## Abstract

Two new DTPA-bis(amide) based ligands conjugated with the arylpiperazinyl moiety were synthesized and subsequently transformed into their corresponding Gd(III) complexes **1** and **2** of the type [Gd(L)H_2_O] nH_2_O. The relaxivity (*R*_1_) of these complexes was measured, which turned out to be comparable with that of Omniscan^®^, a commercially available MRI contrast agent. The cytotoxicity studies of these complexes indicated that they are non-toxic, which reveals their potential and physiological suitability as MRI contrast agents. All the synthesized ligands and complexes were characterized with the aid of analytical and spectroscopic methods, including elemental analysis, ^1^H-NMR, FT-IR, XPS and fast atom bombardment (FAB) mass spectrometry.

## 1. Introduction

Magnetic Resonance Imaging (MRI) is at present one of the most powerful and efficient non-invasive imaging modalities available for clinical diagnosis. MRI is considered the safest diagnostic technique compared to competing radio-diagnostic methods due to the fact it does use harmful high-energy radiation [1,2,3]. With an enormous diagnostic potential, MRI can be used to assess anatomical changes and for monitoring of organ functions, for instance following functions of the human brain on a real time-scale by functional-MRI (fMRI). In cranial abnormalities or multiple sclerosis, MRI is considered the only reliable diagnostic method [4,5]. MRI contrast agents (CAs) usually involve low molecular weight Gd(III) chelates with an acyclic or macrocyclic ligand [6]. They are diagnostic magneto-pharmaceuticals used to enhance the image contrast by increasing the water proton relaxation rate in the body. The efficacy, known as relaxivity, of a CA is measured by its ability to transmit the paramagnetic properties into the bulk water proton and thereby shorten the longitudinal relaxation (*T*_1_) time of water protons, which in turn provides impressive anatomical information [2]. Some representative advantages of employing the Gd(III) ion in most MRI CAs are due to its favorable combination of a large magnetic moment (spin-only μ_eff_ = ¼ 7.94 BM, from seven half-filled f orbitals) and long electron spin relaxation time (10^−8^ to 10^−9^ s, from symmetric S electronic state) [7].

In general, anionic Gd-complexes, for instance Gd(DTPA)^2−^, suffer from limitations such as hyperosmolality under physiological conditions and limited utility in focal lesion detection, leading to adverse effects [8]. To overcome the inherent limitations of anionic Gd-complexes and to improve the tissue and/or organ-specificity, the preparation of neutral Gd-macrocyclic analogues for the development of efficient (“optimized”) CAs is highly desirable [9,10,11,12]. Earlier reports have suggested that incorporation of alkyl and aromatic groups in the side arm of diethylenetriamine pentaacetic acid (DTPA) rendered excellent relaxivity and water solubility [13,14,15]. In the light of the above, we have designed novel ligands from the reaction of DTPA-bis(anhydride) with suitably modified arylpiperazines and subsequently transformed them into their corresponding Gd(III)-complexes **1** and **2**. Herein, we wish to disclose the synthesis, characterization, relaxivity measurements and cytotoxic studies of these new complexes.

## 2. Results and Discussion

The development of an optimum MRI contrast agent would necessitate consideration of the high relaxivity, non-cytotoxicity and high water solubility of the targeted complexes. As suggested by earlier reports, modification of the ligand, for instance by the introduction of polar groups on the alkyl substituents of the amide N-atoms of DTPA-bis(amide), can lead to the formation of water soluble Gd-complexes [16]. Therefore, we intended to synthesize Gd-complexes with different arylpiperazine ligands bearing different functionalities in the aryl moiety.

**Scheme 1:**
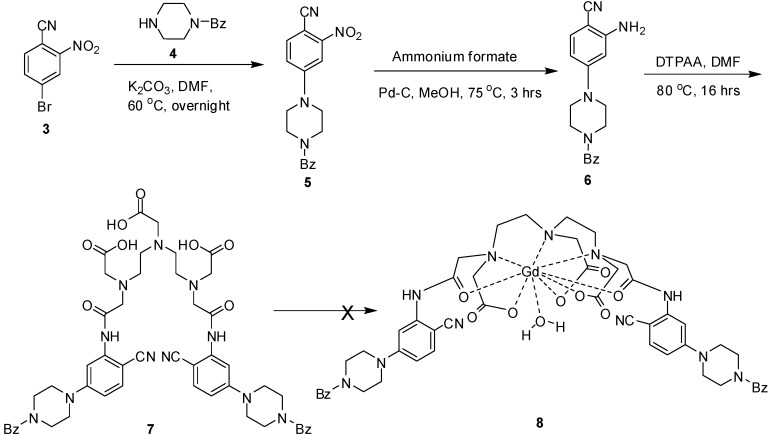
Synthesis of ligand **7**.

To this end, we first planned to synthesize ligand **7** (Scheme 1), possessing a nitrile group in the aryl moiety. Condensation of benzonitrile **3** [17] with piperazine **4** in DMF generated the coupled product **5** in excellent yield. Reduction of the nitro group of intermediate **5**, using Pd-C or Ra-Ni as catalysts, proved problematic and led to the formation of complex mixtures of products, which were difficult to resolve by column chromatography purification. Hence, the reduction of nitro group via transfer hydrogenation with ammonium formate in methanol, using Pd-C as catalyst was employed to produce the corresponding intermediate **6** in high yield. Finally, reaction of aryl amine **6** with diethylene triamine pentaacetic acid dianhydride (DTPAA) [18] in DMF produced the desired ligand **7**. With **7** available, we turned to the synthesis of Gd-complex formation. Unfortunately, the reaction of **7** either with GdCl_3_ or Gd(OAc)_3_ in pyridine was unsuccessful, unreacted **7** being recovered from these reactions. The decreased reactivity of **7** towards complexation could be attributed to both steric and electronic factors. The substitution position and electron withdrawing ability of nitrile group on the aryl moiety generated congestion and reduced the nucleophilicity of the amine moiety (Scheme 1).

**Scheme 2:**
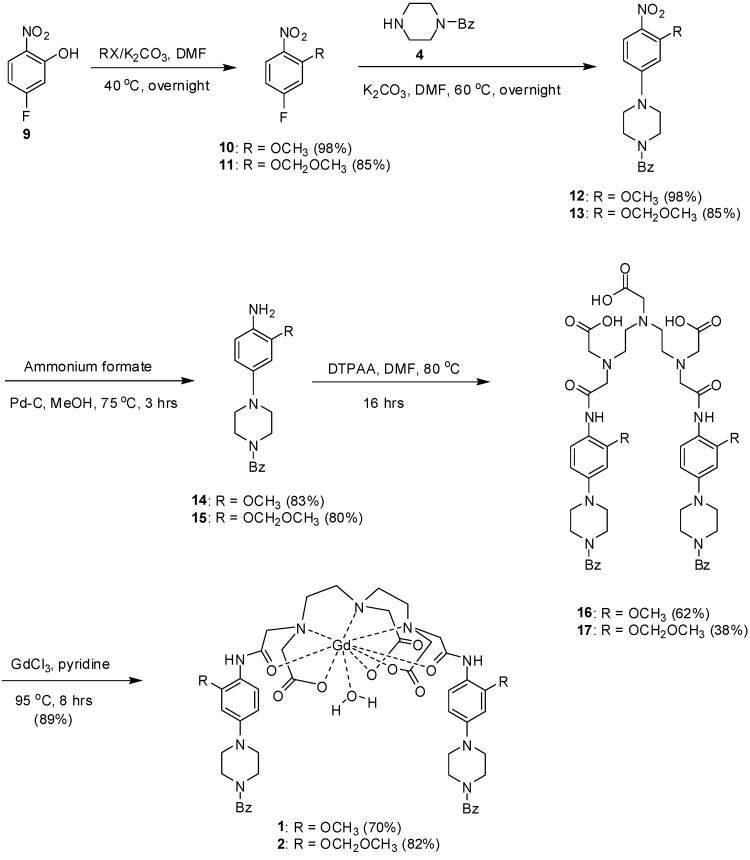
Synthesis of complexes **1** and **2**.

Consequently, we decided to synthesize ligands **16** and **17**, which bear methoxy and methoxymethyl substituents in the aryl moiety (Scheme 2). Thus, phenol **9** was reacted with the appropriate alkyl halide in DMF to produce known compounds **10** [19] and **11** [20], respectively. Treatment of intermediates **10** and **11** with piperazine **4** produced **12** and **13** in good yields (Scheme 2). Reduction of the nitro group of intermediates **12** and **13** with ammonium formate in methnaol, using Pd-C as catalyst, generated the corresponding aryl amines **14** and **15** in high yields. Condensation of amines **14** and **15** with DTPAA produced the desired ligands **16** and **17**, respectively. Finally, heating of ligands **16** and **17** with GdCl_3_ in pyridine produced the desired complexes **1** and **2** in good yield (Scheme 2). The structures of complexes **1** and **2** were established by their infrared (IR) spectra, elemental analysis, fast atomic bombardment (FAB) mass spectrometry and X-ray photoelectron spectroscopy (XPS) measurements. Complexes **1** and **2** are highly hygroscopic and were isolated as hydrated solids. The appearance of the ν (OH) band from the water of crystallization at 3426 and 3480 cm^−1^, supported this observation [13]. The disappearance of the carbonyl stretching bands of the free ligands at 1728 and 1733 cm^−1^ in both complexes **1** and **2** indicated the participation of the carbonyl groups in coordination [21].

**Figure 1:**
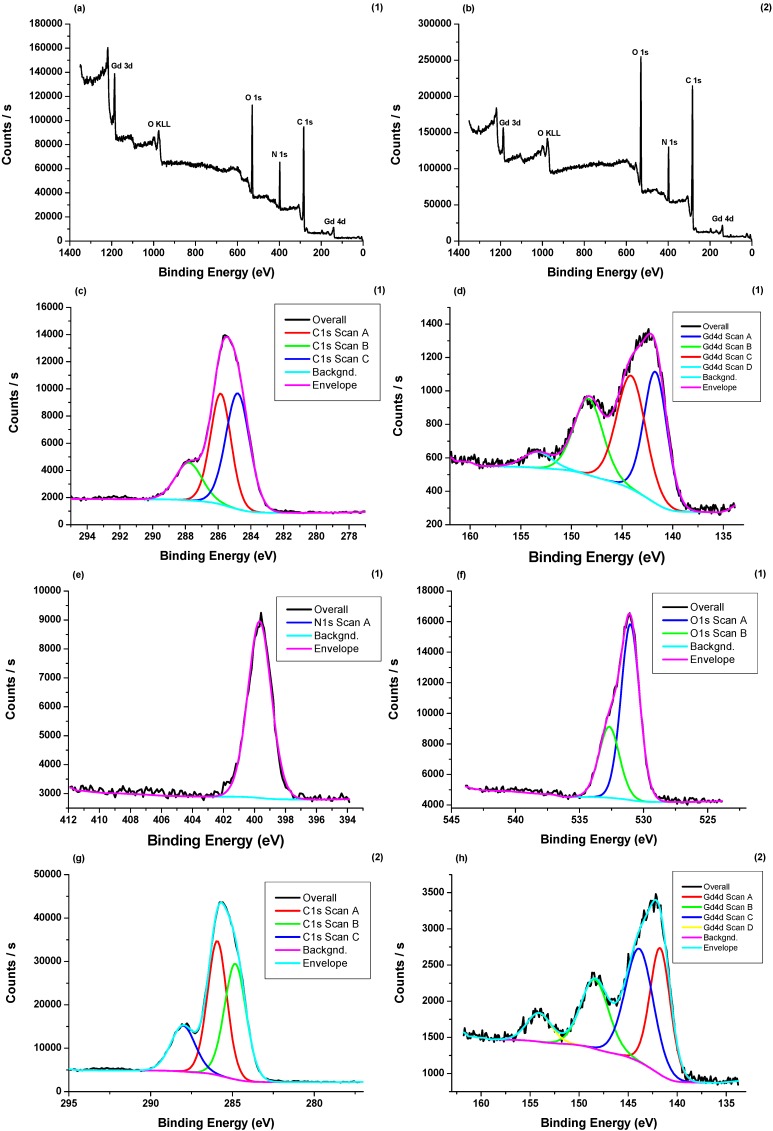

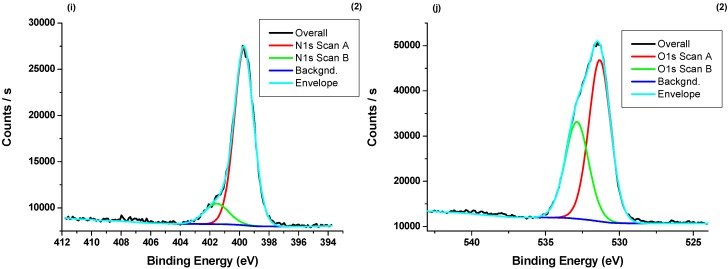
Survey XPS spectrum of **1** (**a**) and **2** (**b**), showing all elements except hydrogens. XPS spectrum of **1**: (**c**) C 1s region, (**d**) Gd 4d region, (**e**) N 1s region and (**f**) O 1s region. **2**: (**g**) C 1s region, (**h**) Gd 4d region, (**i**) N 1s region and (**j**) O 1s region.

The chemical composition of compounds **1** and **2** was further confirmed by XPS (Figure 1). In compound **1**, the C 1s region showed three peaks (eV) at 284.8 (C-C), 285.8 (C-N, C-O) and 287.7 (C=C, C=O) [4], whereas the Gd 4d region had four peaks, showing a multiplet structure, the N 1s region displayed one peak at 399.7 eV. Likewise, the O 1s region has peaks (eV) at 531.0 (C-O) and 532.7 (C=O) (Figure 1) [22]. The XPS measurement of compound **2** showed a similar peak pattern as **1** (Figure 1).

After structural characterization of **1** and **2**, we next moved on to measure their relaxvities. Whereas the commercially available MRI contrast agent Omniscan^®^ is freely soluble in water and methanol; compounds **1** and **2** have moderate water solubility. Relaxvities were calculated as the inverse of the relaxation times per mM based on the earlier report [23]. The R_1_ data (Table 1) revealed that **2** had a higher relaxivity than **1**, but lower than Omniscan^®^. This relatively lower relaxivity of **1** and **2** compared Omniscan^®^ could be attributed to their lower solubility in water.

**Table 1:** Relaxivity and cytotoxicity data of complexes **1** and **2**.

CA	T_1_ (ms)	R_1_ (mM^−1^s^−1^)	*In Vitro* Cytotoxicity (IC_50_ μg/mL)	
**1**	415.72 ± 2.32	2.40	**1**	>50
**2**	360.93 ± 1.56	2.77	**2**	>50
Omniscan^®^^a^		3.2	Cycloheximide	0.13 ± 0.02

^a^ Data obtained from reference [24].

*In vitro* cell toxicity of compounds **1** and **2** was studied on adherent 3T3 mouse embryonic fibroblastic cells by the MTT (3-(4,5-dimethylthiazol-2-yl)-2,5-diphenyltetrazolium bromide) assay [25]. This assay is used as a quantitative colorimetric method to measure cytotoxicity as well as cell viability. The study revealed that compounds **1** and **2** were non-toxic (Table 1), which warrants their physiological suitability as potential contrast agents for MRI.

In conclusion, two new Gd(III) complexes **1** and **2** of the type [Gd(L)H_2_O]·nH_2_O have been synthesized The relaxivity of these complexes was slightly lower compared to Omniscan^®^, a commercially available MRI contrast agent. The lower relaxivity of **1** and **2** was attributed to their lower solubility in water. The cytotoxicity studies of these complexes revealed that they are non-toxic which warrants their potential and physiological suitability as MRI contrast agents. The water solubility of these compounds could be increased by introducing polar functions in the aromatic ring, which in turn, may improve their relaxivity. Hence, these compounds may serve as a starting point to obtain optimized structures to produce more efficient MRI contrast agents.

## 3. Experimental Section

### 3.1. General Information

Melting points were determined on a Büchi apparatus (Büchi, Flawil, Switzerland) and were uncorrected. Elemental analysis was carried out on a Perkin Elmer Elemental Analyzer Series 11 Model 2400 (PerkinElmer, Waltham, MA, USA). IR spectra were recorded on a Perkin Elmer 16F PC FTIR spectrophotometer. ^1^H- and ^13^C-NMR spectra were measured in CDCl_3_ and DMSO-*d*_6_ using TMS as internal standard on a LA 500 MHz spectrometer (JEOL, Peabody, MA, USA). Mass spectra were recorded on a 6890 N GC–MS system (Agilent Technologies, Santa Clara, CA, USA). Analytical TLC was carried out on silica gel 60 F_254_ plates (catalog #-5554-7, Merck, Darmstadt, Germany); column chromatography was carried out on Merck silica gel (200–400 mesh, catalog # 61860805001730). All chemicals and reagents were obtained from Sigma-Aldrich (Buchs, Switzerland) in reagent grade and were used without further purification.

### 3.2. Relaxivity Measurements

Longitudinal relaxation times measurements were performed in a 3T MRI machine (3T Imaging, Morton Grove, IL, USA) The magnetic resonance images were taken at 17 different TR values ranging from 20 to 1500 ms and the T_1_ were obtained from non-linear least square fit measured at each T_1_ values. R_1_ were calculated as an inverse of relaxation times per mM.

### 3.3. In Vitro Cell Toxicity

Toxicity for compounds **1** and **2** was analyzed on adherent 3T3 mouse embryonic fibroblastic cells by MTT assay following Mesaik *et al.* [25] with some modification. In brief, cells were incubated at 6 × 104 mL^−1^ concentration in 96 well flat bottom plate in 5% CO_2_ and 37 °C for 24 h. After adherence of cells, compounds were added at various concentrations for further 48 h incubation. On day 3 the tetrazolium dye MTT was added. After 4 h of incubation media was removed and organic solvent DMSO was added to dissolve insoluble purple formazan. Absorbance was taken at 540 nm using a spectrophotometer and the % viability of the cells was calculated.

### 3.4. X-ray Photoelectron Spectroscopy (XPS) Measurements

The chemical composition of compounds **1** and **2** was studied by XPS. The experiment was performed in a Escalab 250Xi spectrometer (Thermo Scientific, West Palm Beach, FL, USA) equipped with a monochromated Al *K_α_* (1486.6 eV) X-ray source. A low-energy electron flood gun was used for surface charge compensation. The spectrometer energy was calibrated by fixing Cu 2p_3/2_, Ag 3d_5/2_ and Au 4f_7/2_ peaks at binding energies of 932.6, 368.2 and 83.9 eV, respectively [22]. The electron energy analyzer was operated in constant pass energy of 30 eV and the electron take off angle was 90°. The instrumental energy resolution was 0.5 eV with X-ray spot size of 650-μm diameter. The base pressure in the analysis chamber was 5.0 × 10^−10^ mbar. The spectra were referenced with C-C 1s peak at 284.8 eV. Avantage software was used for all data processing.

### 3.5. Chemistry

*4-(4-Benzoylpiperazin-1-yl)-2-nitrobenzonitrile* (**5**): To a solution of amine **4** (1.82 g, 9.56 mmol) in DMF (20 mL) at 0 °C was added K_2_CO_3_ (3.61 g, 26.13 mmol) followed by the addition of compound **3** (1.96 g, 8.67 mmol) and the reaction mixture was stirred overnight at 60 °C. After completion of the reaction (TLC analysis), the mixture was cooled to room temperature and diluted with ethyl acetate (75 mL). The solution was washed with H_2_O (30 mL × 3), brine (20 mL × 2) and the organic layer was separated, dried over Na_2_SO_4_ and evaporated under vacuum to afford the title compound **5** as as light yellow amorphous solid (2.16 g, 74%), m.p. 196–197 °C. IR (KBr): 3063, 2999, 2929, 2233, 1670, 1622, 1596, 1578, 1457, 1430, 1389, 1341, 1294, 1249, 1155, 1094, 1072 cm^−1^. ^1^H-NMR (CDCl_3_): δ 3.47 (br. s, 4H, -CH_2_NCH_2_-), 3.79 (br. s, 4H, -CH_2_NCH_2_-), 6.99 (dd, 1H, *J* = 2.6, 9.4 Hz, Ar-H), 7.14 (d, 1H, *J* = 2.7 Hz, Ar-H), 7.40–7.48 (m, 5H, Ar-H), 8.19 (d, 1H, *J* = 9.5 Hz, Ar-H). ^13^C-NMR (CDCl_3_): δ 46.44, 46.84, 110.05, 115.66, 118.89, 126.93, 127.10, 127.79, 128.67, 130.33, 134.69, 137.79, 153.10, 170.61. Anal. Calcd for C_18_H_16_N_4_O_3_: C, 64.28; H, 4.79; and N, 16.66. Found: C, 64.22; H, 4.84; and N, 16.60.

*2-Amino-4-(4-benzoylpiperazin-1-yl)benzonitrile* (**6**): To a solution of compound **5** (2.93 g, 7.12 mmol) in anhydrous methanol (50 mL) was sequentially added Pd-C (10% wet basis, 0.25 g) and ammonium formate (2.25 g, 35.63 mmol) and the mixture was refluxed for 3 h. The reaction mixture was filtered through a pad of Celite and the filtrate was concentrated under vacuum. Column chromatography of the dark purple oily material eluting with MeOH-CH_2_Cl_2_ (1:9) afforded the title compound **6** as a light brown thick oil (1.77 g, 81%). IR (KBr): 3455, 3348, 3060, 2916, 2821, 2213, 1669, 1631, 1577, 1505, 1437, 1388, 1313, 1284, 1241, 1158, 1095 cm^−1^. ^1^H-NMR (CDCl_3_): δ 2.86 (br. s, 4H, -CH_2_NCH_2_-), 3.49 (br. s, 2H, -CH_2_N), 3.83 (br. s, 2H, -CH_2_N), 4.32 (br. s, 2H, -NH_2_), 6.61 (d, 1H, *J* = 8.6 Hz, Ar-H), 6.79 (d, 1H, *J* = 2.4 Hz, Ar-H), 6.94 (dd, 1H, *J* = 2.4, 8.6 Hz, Ar-H), 7.34 (m, 5H, Ar-H). ^13^C-NMR (CDCl_3_): δ 41.81, 47.34, 50.62, 53.32, 95.69, 116.52, 117.54, 119.43, 125.63, 126.76, 128.28, 129.61, 135.19, 142.64, 144.71, 170.09. Anal. Calcd for C_18_H_18_N_4_O: C, 70.57; H, 5.92; and N, 18.29. Found: C, 70.50; H, 5.97; and N, 18.22.

*2,2'-(2,2'-(Carboxymethylazanediyl)bis(ethane-2,1-diyl)bis((2-(5-(4-benzoylpiperazin-1-yl)-2-cyanophenylamino)-2-oxoethyl)azanediyl))diacetic acid* (**7**): To a solution of DTPAA (0.175 g, 0.49 mmol) in DMF (10 mL) was added compound **6** (0.30 g, 0.98 mmol) and the reaction mixture was stirred at 80 °C for 16 h. The mixture was cooled to ~40 °C, filtered through a pad of silica gel and the filtrate was concentrated under reduced pressure. The residue was added dropwise to cold acetone (50 mL) and the precipitated product was filtered by suction, dried under reduced pressure to afford compound **7** as an off-white powder (0.31 g, 65%), m.p. 158–160 °C. IR (KBr): 3467, 2917, 2509, 2362, 2228, 1690, 1627, 1517, 1437, 1389, 1284, 1241, 1160, 1048, 1011, 962, 829, 788, 708, 633, 559, 493, 424 cm^−1^. ^1^H-NMR (DMSO-*d*_6_): δ 2.90–3.20 (m, 10H), 3.43–3.72 (m, 24H), 7.22–7.26 (m, 4H, Ar-H), 7.45–7.49 (m, 14H, Ar-H), 10.03 (s, 2H). ^13^C-NMR (DMSO-*d*_6_): δ 41.21, 48.05, 51.13, 52.46, 55.07, 57.89, 107.65, 117.08, 118.42, 121.01, 125.85, 127.02, 128.49, 129.68, 121.57, 135.72, 147.91, 169.10, 170.01, 172.63. Anal. Calcd for C_50_H_55_N_11_O_10_·H_2_O: C, 60.78; H, 5.81; and N, 15.59. Found: C, 60.72; H, 5.85; and N, 15.52. FAB-MS (*m/z*): calcd for C_50_H_55_N_11_O_10_, 969.4 ([MH]^+^). Found: 969.3 ([MH]^+^).

*(4-(3-Methoxy-4-nitrophenyl)piperazin-1-yl)(phenyl)methanone* (**12**): Following the same procedure adopted for the synthesis of **5**, the reaction of amine **4** with compound **10** (1.49 g, 8.71 mmol) afforded the title compound **12** as as bright yellow amorphous solid (2.91 g, 98%), m.p. 185–187 °C. IR (KBr): 3021, 1627, 1575, 1488, 1461, 1436, 1383, 1336, 1314, 1245, 1156, 1102, 1080 cm^−1^. ^1^H-NMR (DCl_3_): δ 3.40–3.50 (br. s, 4H, -CH_2_NCH_2_-), 3.94 (s, 3H, -OCH_3_), 4.64 (br. s, 4H, -CH_2_NCH_2_-), 6.33 (d, 1H, *J* = 2.4 Hz, Ar-H), 6.43 (dd, 1H, *J* = 2.4, 9.1 Hz, Ar-H), 7.45 (m, 5H, Ar-H), 8.00 (d, 1H, *J* = 9.1 Hz, Ar-H). ^13^C-NMR (CDCl_3_): δ 47.01, 56.23, 97.62, 105.75, 127.08, 128.61, 128.74, 130.16, 134.97, 155.23, 156.07, 170.55. Anal. Calcd for C_18_H_19_N_3_O_4_: C, 63.33; H, 5.61; and N, 12.31. Found: C, 63.26; H, 5.66; and N, 12.26.

*(4-(3-(Methoxymethoxy)-4-nitrophenyl)piperazin-1-yl)(phenyl)methanone* (**13**): Following the same procedure adopted for the synthesis of **5**, the reaction of amine **4** with compound **11** (4.10 g, 21.39 mmol) afforded the title compound **13** as light yellow solid (7.34 g, 97%), m.p. 189–190 °C. IR (KBr): 3032, 1675, 1620, 1575, 1490, 1461, 1436, 1383, 1336, 1314, 1249, 1150, 1102, 1078, 1035 cm^−1^. ^1^H-NMR (CDCl_3_): δ 3.31–3.33 (br. s, 4H, -CH_2_NCH_2_-), 3.45 (s, 3H, -OCH_3_), 3.85 (br. s, 4H, -CH_2_NCH_2_-), 5.21 (s, 2H, -OCH_2_O-), 6.42 (dd, 1H, *J* = 2.4, 9.4 Hz, Ar-H), 6.56 (d, 1H, *J* = 2.4 Hz, Ar-H), 7.36 (m, 5H, Ar-H), 7.85 (d, 1H, *J* = 9.3 Hz, Ar-H). ^13^C-NMR (CDCl_3_): δ 46.99, 56.53, 67.02, 95.38, 101.37, 106.83, 126.91, 128.24, 128.42, 129.92, 134.87, 153.48, 154.77, 162.33, 170.28. Anal. Calcd for C_19_H_21_N_3_O_5_: C, 61.45; H, 5.70; and N, 11.31. Found: C, 61.41; H, 5.75; and N, 11.25.

*(4-(4-Amino-3-methoxyphenyl)piperazin-1-yl)(phenyl)methanone* (**14**): Following the same procedure adopted for the synthesis of **6**, the reduction of the nitro group of compound **12** (2.43 g, 7.12 mmol) afforded compound **14** as a dark brown thick oil (1.84 g, 83%). IR (KBr): 3447, 3346, 3058, 2917, 1673, 1629, 1518, 1438, 1386, 1323, 1282, 1245, 1197, 1170, 1093, 1034, 1015 cm^−1^. ^1^H-NMR (CDCl_3_): δ 2.92 (br. s, 2H, -CH_2_N), 3.07 (br. s, 2H, -CH_2_N), 3.53 (br. s, 2H, -CH_2_N), 3.79 (s, 3H, -OCH_3_), 3.89 (br. s, 2H, -CH_2_N), 6.37 (dd, 1H, *J* = 2.4, 8.2 Hz, Ar-H), 6.47 (d, 1H, *J* = 2.4 Hz, Ar-H), 6.59 (d, 1H, *J* = 8.3, Ar-H), 7.38 (m, 5H, Ar-H). ^13^C-NMR (CDCl_3_): δ 42.23, 47.80, 51.59, 51.81, 53.36, 55.35, 102.74, 109.98, 115.21, 126.96, 128.39, 129.64, 130.85, 135.56, 144.25, 147.85, 170.24. Anal. Calcd for C_18_H_21_N_3_O_2_: C, 69.43; H, 6.80; and N, 13.49. Found: C, 69.36; H, 6.87; and N, 13.42.

*(4-(4-Amino-3-(methoxymethoxy)phenyl)piperazin-1-yl)(phenyl)methanone* (**15**): Following the same procedure adopted for the synthesis of **6**, the reduction of the nitro group of compound **13** (1.53 g, 4.12 mmol) afforded compound **15** as a dark purple thick oil (1.13 g, 80%). IR (KBr): 3447, 3352, 3010, 2953, 1671, 1628, 1516, 1436, 1366, 1325, 1284, 1241, 1214, 1150, 1074 cm^−1^. ^1^H-NMR (CDCl_3_): δ 2.92 (br. s, 2H, -CH_2_N), 3.08 (br. s, 2H, -CH_2_N), 3.46–3.44 (m, 5H, -OCH_3_, -CH_2_N), 3.53 (br. s, 2H, -CH_2_N), 3.89 (br. s, 2H, -NH_2_), 5.13 (s, 2H, -OCH_2_O-), 6.44 (dd, 1H, *J* = 2.4, 8.3 Hz, Ar-H), 6.61 (d, 1H, *J* = 8.4 Hz, Ar-H), 6.72 (d, 1H, *J* = 2.4 Hz, Ar-H), 7.38 (m, 5H, Ar-H). ^13^C-NMR (CDCl_3_): δ 42.18, 47.69, 51.40, 55.99, 95.25, 106.35, 111.64, 115.81, 126.95, 128.37, 129.63, 131.22, 135.53, 144.12, 145.52, 170.23. Anal. Calcd for C_19_H_23_N_3_O_3_: C, 66.84; H, 6.79; and N, 12.31. Found: C, 66.77; H, 6.86; and N, 12.24.

*2,2'-(2,2'-(Carboxymethylazanediyl)bis(ethane-2,1-diyl)bis((2-(4-(4-benzoyl-piperazin-1-yl)-2-methoxyphenylamino)-2-oxoethyl)azanediyl))diacetic acid* (**16**): Following the same procedure adopted for the synthesis of **7**, condensation of compound **14** (0.89 g, 2.86 mmol) with DTPAA afforded compound **16** as a white powder (0.87 g, 62%), m.p. 151–153 °C. IR (KBr): 3453, 3021, 2924, 1728, 1629, 1531, 1441, 1389, 1285, 1201, 1156, 1089, 1031, 1013 cm^−1^. ^1^H-NMR (DMSO-*d*_6_): δ 2.71 (br. s, 4H), 2.96–3.30 (m, 6H), 3.34–3.73 (m, 24H), 3.75 (s, 6H, -OCH_3_), 6.40 (dd, 2H, *J* = 2.4, 8.2 Hz, Ar-H), 6.60 (d, 2H, *J* = 2.4 Hz, Ar-H), 7.44 (m, 10H, Ar-H), 7.93 (d, 2H, *J* = 8.2 Hz, Ar-H), 9.34 (s, 2H). ^13^C-NMR (DMSO-*d*_6_): δ 30.97, 49.39, 52.54, 55.17, 55.70, 56.01, 100.77, 107.72, 119.85, 121.68, 127.26, 128.82, 130.01, 135.91, 168.99, 169.59, 172.70. Anal. Calcd for C_50_H_61_N_9_O_12_·H_2_O: C, 60.17; H, 6.36; and N, 12.63. Found: C, 60.11; H, 6.42; and N, 12.57. FAB-MS (*m/z*): calcd for C_50_H_61_N_9_O_12_, 979.44 ([MH]^+^). Found: 979.3 ([MH]^+^).

*2,2'-(2,2'-(Carboxymethylazanediyl)bis(ethane-2,1-diyl)bis((2-(4-(4-benzoyl-piperazin-1-yl)-2-(methoxymethoxy)phenylamino)-2-oxoethyl)azanediyl))diacetic acid* (**17**): Following the same procedure adopted for the synthesis of **7**, condensation of compound **15** (0.26 g, 0.76 mmol) with DTPAA afforded compound **17** as a white powder (0.15 g, 38%), m.p. 165–167 °C. IR (KBr): 3452, 3021, 2929, 1722, 1626, 1536, 1443, 1389, 1285, 1201, 1153, 1089, 1031, 1015 cm^−1^. ^1^H-NMR (DMSO-*d*_6_): δ 2.74–2.82 (br. s, 4H), 2.95–3.13 (m, 6H), 3.29–3.70 (m, 24H), 3.73 (s, 6H, -OCH_3_), 5.19 (s, 4H, -OCH_2_O-), 6.54 (dd, 2H, *J* = 2.6, 8.6 Hz, Ar-H), 6.72 (d, 2H, *J* = 2.5 Hz, Ar-H), 7.44 (m, 10H, Ar-H), 7.92 (d, 2H, *J* = 8.5 Hz, Ar-H), 9.50 (s, 2H). ^13^C-NMR (DMSO-*d*_6_): δ 49.09, 52.36, 55.89, 59.00, 94.54, 103.68, 109.01, 120.84, 126.99, 128.45, 129.59, 135.81, 147.10, 169.04. Anal. Calcd for C_52_H_65_N_9_O_14_·H_2_O: C, 59.02; H, 6.38; and N, 11.91. Found: C, 58.96; H, 6.44; and N, 11.85. FAB-MS (*m/z*): calcd for C_52_H_65_N_9_O_14_, 1039.47 ([MH]^+^). Found: 1039.3 ([MH]^+^).

*Synthesis of Complex* (**1**): To a solution of compound **16** (0.30 g, 0.31 mmol) in pyridine (15 mL) was added GdCl_3_·6H_2_O (0.11 g, 0.30 mmol) and the reaction mixture was stirred at 90 °C for 8 h. The mixture was filtered through a pad of Celite while hot and the filtrate was concentrated under reduced pressure. The residue was added dropwise to cold acetone (25 mL) and the precipitated product was filtered, dried under reduced pressure to afford compound **1** as a white amorphous solid (0.25 g, 70%), m.p. > 300 °C. IR (KBr): 3426, 3036, 2973, 1641, 1438, 1324, 1251, 1202, 1153, 1092, 1007 cm^−1^. Anal. Calcd for C_50_H_58_GdN_9_O_12_·4H_2_O: C, 49.78; H, 5.51; and N, 10.45. Found: C, 49.71; H, 5.57; and N, 10.38. FAB-MS (*m/z*): calcd for C_50_H_58_GdN_9_O_12_, 1134.34 ([MH]^+^). Found: 1134.3 ([MH]^+^).

*Synthesis of Complex* (**2**): Following the same procedure adopted for the synthesis of **1**, condensation of ligand **17** (0.15 g, 0.14 mmol) with GdCl_3_·6H_2_O afforded complex **2** as a light purple amorphous solid (0.14 g, 82%). m.p. > 300 °C. IR (KBr): 3480, 3042, 2914, 1631, 1437, 1322, 1245, 1153, 1090 cm^−1^. Anal. Calcd for C_52_H_62_GdN_9_O_14_·4H_2_O: C, 49.32; H, 5.57; and N, 9.95. Found: C, 49.25; H, 5.64; and N, 9.88. FAB-MS (*m/z*): calcd for C_52_H_62_GdN_9_O_14_, 1194.37 ([MH]^+^). Found: 1194.3 ([MH]^+^).

## 4. Conclusions

In conclusion, two new Gd(III) complexes **1** and **2** of the type [Gd(L)H_2_O]·nH_2_O have been synthesized The relaxivity of these complexes were slightly lower compare to Omniscan^®^, a commercially available MRI contrast agent. The lower relaxivity of **1** and **2** was attributed to their lower solubility in water. The cytotoxicity studies of these complexes revealed that they are non-toxic which warrant their potential and physiological suitability as MRI contrast agents. The water solubility of these complexes may be increased by introducing polar functions in the aromatic ring, which in turn, may improve their relaxivity. Hence, these complexes may serve as a starting point to get optimized compound to achieve more efficient MRI contrast agent.
